# Percutaneous occlusion of post-myocardial infarction ventricular septum rupture

**DOI:** 10.1007/s12471-013-0498-4

**Published:** 2013-11-28

**Authors:** F. Risseeuw, I. Diebels, T. Vandendriessche, D. De Wolf, I. E. Rodrigus

**Affiliations:** 1 Department of Cardiac Surgery, Antwerp University Hospital, Wilrijkstraat 10, 2650 Edegem, Belgium; 2 Department of Cardiology, Antwerp University Hospital, Wilrijkstraat 10, 2650 Edegem, Belgium; 3 Department of Paediatrics (Paediatric Cardiology), Antwerp University Hospital, Wilrijkstraat 10, 2650 Edegem, Belgium

**Keywords:** Post infarction ventricular septal rupture, Occluder device, Acute myocardial infarction, Transcatheter, Percutaneous, Systematic review

## Abstract

**Aims:**

The aim of this systematic review is to gain insight into the published experience on percutaneous closure of a post-infarction ventricular septal rupture (VSR).

**Method:**

Relevant literature was obtained by MeSH-term searches in the online search-engine PubMed. Articles published in the last 10 years were included. Further filtering was done by using search limits and individual article selection based on the aims of this systematic review.

**Conclusion:**

Percutaneous closure is a potential technique in a select group of patients. The presence of cardiogenic shock and closure in the acute phase after VSR diagnosis are important risk factors of mortality. Device implantation is in general successful with few procedure-related complications. Reduction of the shunt fraction has been reported frequently. This technique is a less invasive alternative to surgical treatment and should be applied on a case-by-case basis.

## Introduction

Post-infarction ventricular septum rupture (VSR) is a rare mechanical complication of an acute myocardial infarction (AMI). Due to the use of aggressive antithrombotic medication and more efficient revascularisation therapy, the incidence of post-infarction VSR has decreased from 1–2 % to 0.25–0.31 % [1]. An acute left-to-right shunt following a VSR may lead to acute haemodynamic deterioration. When conservative treatment is applied, mortality rates are as high as 87–100 % [2, 3]. Unfortunately, even surgical patch repair has a high mortality of 47 % in the first 30 days after closure [2]. To date, the American College of Cardiology and American Heart Association (ACC/AHA) still advise immediate surgical closure of the VSR, often combined with (multiple) coronary artery bypass grafting [4]. Due to the high mortality rate, less invasive alternative treatments, such as the use of percutaneous occluders, have been investigated. This article gives an overview of recently published experiences with the use of percutaneous occluders as a treatment for VSR.

## Search strategy

For this systematic review we used the search engine PubMed to browse through the online database MEDLINE using the following MeSH terms: [ventricular septal rupture] OR [ventricular septal defect] AND [myocardial infarction] AND [septal occluder device] OR [transcatheter] OR [percutaneous]. English case series published from 2002 onwards were included. The last search took place in February 2013. Only case series of ≥4 patients were included as we believed they were less sensitive to a selection bias than pure case reports. Other relevant articles were also included.

## Procedure

The technique of percutaneous closure of a VSR is based upon the well proven and widely used percutaneous technique for closing a congenital ventricular septal defect. Echocardiography with colour Doppler is used to determine the size and anatomy of the VSR. More detailed information about the lesion could be obtained by other imaging methods, such as computed tomography, but up till now only few case reports have been published [5]. Indication for a surgical or a percutaneous closing technique should be on a case-by-case basis. Selection of the occlusion device will be discussed later in this article.

Cannulation of the femoral artery and femoral vein or internal jugular vein is performed using the Seldinger technique. A guidewire is introduced into the artery, through the aortic valve and is advanced through the VSR into the right ventricle and pulmonary artery. A second snaring wire is introduced through the vein to connect to the guidewire in the pulmonary artery. By retracting the snared wires, the guidewire now forms an arteriovenous (AV) loop.

An alternative method is by creating a venovenous (VV) loop. In this method the cannulae are inserted in the femoral and jugular vein. The initial guidewire, inserted via de femoral vein, perforates through the atrial septum into the left heart system. It then travels via the VSR into the right ventricle and will be snared in the pulmonary artery. The procedure is at that point the same as with the AV-loop method.

The delivery sheath is advanced from the venous side loop over the guidewire through the VSR into the left ventricle. Using fluoroscopy and/or echocardiography, correct positioning of the delivery sheath is confirmed. The guidewire is then retracted leaving the delivery sheath in position. Once echocardiographic confirmation of the necessary device size has been achieved, the device is placed inside its catheter and advanced through the VSR using the delivery sheath. The distal disc is opened, the device is retracted, so that it will be secured against the septal tissue at the side of the left ventricle. The second proximal disc is opened by further retracting the delivery sheath. Correct positioning of the device and closure is confirmed by echocardiography and/or fluoroscopy. If placement is satisfactory, the occluder is released. The procedure is concluded by retracting the delivery sheath, guidewire and cannulae.

## VSR localisation and characteristics

An anterior or inferior AMI may lead to VSR formation due to progressive septal tissue necrosis. The VSR diameter will often increase over time until stabilisation, due to the scarring of the surrounding tissue. Localisation is also an important factor as an apical or perivalvular VSR might not be suitable for percutaneous occlusion. When treating a perivalvular VSR the device may interfere with valvular components [6, 7]. Due to the presence of the ventricle free walls, an apically located VSR might pose difficulties in correctly positioning the device, thus making these VSR localisations more challenging [8].

**Fig. 1:**
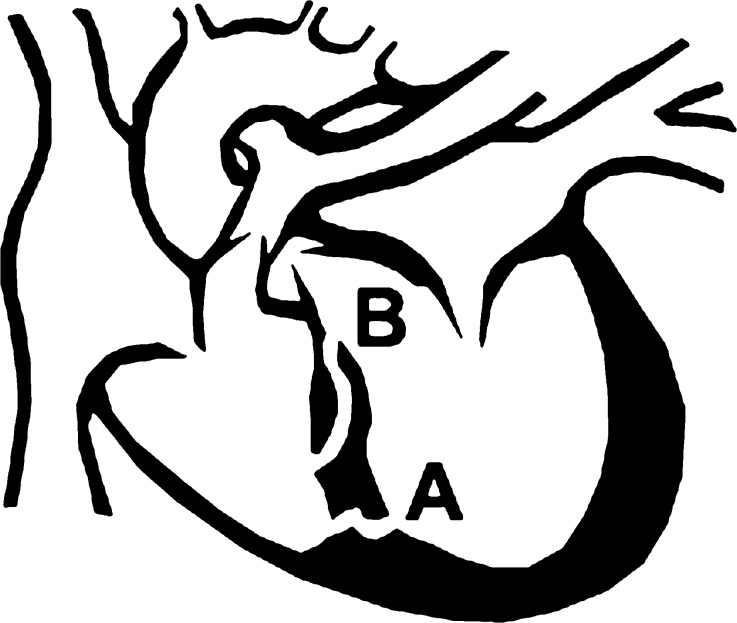
Simple (**a**) versus complex (**b**) ventricular septal rupture

The characteristics of a VSR greatly differ from those of a congenital VSD. A congenital VSD is often symmetrical, has strong borders and a relatively straight connection between the left and right ventricle. In contrast, a VSR forms due to acute ischaemic damage of the septum. As a result, a VSR is an asymmetrical tear with unstable necrotic borders. In post-mortem research VSR have been classified into simple versus complex ruptures (Fig. 1). Simple VSRs are straight horizontal septal canals whilst complex VSRs travel serpiginously through the septum before exiting at a different level. The majority of complex VSRs were the result of inferior AMI [9].

Percutaneous closure of a complex VSR is obviously more challenging, but once a correct AV loop or VV loop is achieved, device placement is possible.

## Occluder devices

Based on the existing literature, a variety of devices for percutaneous closure of a VSR have been used. These devices are the atrial-septal-defect occluder (ASDo), muscular-ventricular-septal-occluder (mVSDo) and recently a specific post-infarction mVSDo occluder (PimVSDo) developed by Amplatzer. The diameters of the applied devices were, on purpose, significantly larger than the diameter of the VSR measured using different imaging techniques. This ‘oversizing’ was particularly important when closures were attempted in the acute phase. Some authors even stated that the optimal diameter should be twice the size of the measured VSR diameter or at least 10 mm larger [10, 11]. This prevents incomplete closure or dislodging and embolisation of the device due to continued septal necrosis. Occlusion in the chronic phase demanded occluding devices that were only moderately larger in diameter than the measured VSR. A device sized 4–7 mm larger than the VSR should suffice [12].

It is not yet certain which occluder device is the best option for the treatment of a VSR. However, only the use of ASDo was not the best treatment option, especially in the acute phase. Due to the fact that the combination of a high pressure gradient between the two ventricles and the high permeability of an ASDo, it was unable to provide sufficient occlusion of the VSR [2]. Some authors preferred the specific Amplatzer PimVSDo. This device has a wider waist, larger disks and a denser construction, which should lead to a faster occlusion over a wider septal region. It is important to note that VSR were often accompanied by accessory VSR. Therefore larger discs were more likely to cover any accessory VSR [12–14]. Apart from the technical aspects, the personal preference of the treating physician will also affect the decision as to which occluder device will be used during the procedure.

## Interval between diagnosis and occlusion

As mentioned earlier, the acute necrotic septum forms an inadequate base for surgical patch repair. Therefore, despite ACC/AHA guidelines, there is a tendency to defer surgical treatment by 2–4 weeks. In this time the necrotic process will stabilise and scarring of the surrounding tissue will occur, which will form a better fundament for a successful fixation of the patch. The decision as to whether deferring is feasible or not should be based on the haemodynamic stability of the patient. Patients will typically be treated with supportive medication, an intra-aortic balloon pump (IABP) and/or mechanical circulatory support (MSC) [11, 15].

Acute-phase occlusion was defined as closure within <14 days after diagnosis of the VSR. In the acute phase, patients are often critically ill and have high logistic EuroSCORES [16]. Despite high procedural success (50–86.2 %), haemodynamic stabilisation is often not achieved, leading to a very high residual mortality of 42–100 % [11, 16–19].

Treatment during the chronic phase was defined as ≥14 days after diagnosis of the VSR. Occlusion was performed on haemodynamically more stable patients, weeks or even months after VSR formation. Procedural success is high (71.4–100 %) and coincides with a lower mortality rate of 20–38.9 % [12, 20–22]. Table 1 summarises the included case series.

**Table 1:** Procedure characteristics

Author, publicationyear	VSR patients	Acute phase (<14 d)	Chronic phase (≥ 14 d)	Time between VSR diagnosis and occlusion	Succesful device placement	Mortality
Assenza et al. (2013) [23]	30	–	–	Med: 27 d	–	7/30
Maltais et al.(2009) [11]	12	12	0	Mean: 6,1 d	–	5/12
Thiele et al. (2009) [17]	29	29	0	Mean: CS: 1 d	25/29	CS: 13/14
				Mean: Non-CS: 3 d		Non-CS: 4/11
Ahmed et al. (2008) [18]	5	2	3	Med: 50 d	4/5	2/5
Martinez et al. (2007) [10]	5	3	2	Mean: 6	4/5	1/5
Marinakis et al. (2007) [16]	8	6	2	–	–	7/8
Bialkowski et al. (2007) [19]	17	0	19	Mean: 8,9 w	14/19	3/19
Demkow et al. (2005) [22]	11	1	10	Mean: 15,4 d	10/11	3/11
Holzer et al. (2004) [24]	18	6	12	Med: 25 d	16/18	7/18
Szkutnik et al. (2003) [12]	7	0	7	Mean: 7,7 w	5/7	1/7
Goldstein et al. (2003) [20]	4	0	4	Mean: 58 d	3/4	1/4
Marshall et al. (2002) [21]	18	0	>10	–	–	7/18
Chessa et al. (2002) [25]	12	3	9	–	10/12	–
Total	176	62	78	–	91/110 (82.7 %)	61/162 (37,7 %)

*AMI* acute myocardial infarction, *VSR* ventricular septum rupture, *Med* median, *CS* cardiogenic shock

## Cardiogenic shock

Patients suffering from a VSR will frequently present with cardiogenic shock (CS) due to an acute left-to-right shunt complicating the initial AMI. The presence of CS is associated with a tremendous increase in mortality. In the prospective research of Thiele et al. [17], all VSRs were treated directly after VSR diagnosis independent of the haemodynamic state. The acute-phase mortality, in this specific research defined as closure within 30 days after VSR diagnosis, in the CS population was 86 % (n = 12/14) increasing to 93 % (n = 13/14) in the chronic phase. In contrast, the non-CS population had a mortality rate of 36 % (n = 4/11) in the acute phase, which remained the same in the chronic phase. This case series was particularly useful due to the fact that all patients were directly treated when the VSR was diagnosed, independent of haemodynamic stability. This rules out any selection bias based on treatment delay and shows the importance of controlling CS before attempting VSR occlusion [17].

## Indications

Percutaneous occlusion is a less invasive alternative to surgical treatment, thus making it especially valuable when surgical closure is not indicated. It is important to state that percutaneous occlusion is an option in every case where the decision to perform an intervention has been made, because transformation to the classic surgical approach is possible at any time.

This technique is most successful when performed in the chronic phase after post-infarction VSR diagnosis. If shunts remain or residual shunts form, placement of multiple devices is possible. Percutaneous occlusion is also successful in patients who present with clinically significant residual shunting in follow-up after initial successful closure [17, 21–23]. Occlusion in the acute phase in haemodynamic unstable patients had the aim of permanently closing the VSR or stabilising the patient as a step-up approach to surgical treatment. Unfortunately results were not clearly beneficial compared with surgery. Percutaneous treatment of a VSR should always be considered when a patient refuses surgical treatment.

## Complications

A VSR differs greatly from a congenital VSD. The mechanism of origin of the VSR often results in a more complex anatomy, making complete closure difficult. Apart from that, the device has to be placed in fragile necrotic tissue where every manipulation can lead to an increase of the VSR diameter and device displacement. Persistent left-to-right shunting can be caused by progressive septal necrosis or by complications in relation to device placement. The presence of such a residual shunt differs greatly amongst the different authors (12.5–100 %) [10–12, 18, 20].

When percutaneous treatment was performed, a variety of procedure-related complications occurred: device embolisation, left ventricle free wall rupture, cardiac arrhythmia, haematoma at the puncture side, haemolysis and dislodging of the occluding device. Procedure-related complications did not occur frequently and were rarely the direct cause of mortality.

Complications not related to the procedure were multiple organ failure [11, 12, 16, 18], haemolytic anaemia [18, 24, 25] and sepsis [16]. Mortality occurring in the long-term period following intervention was often the result of one of these complications.

## Conclusion

Percutaneous closure of a VSR is a feasible and a relatively safe technique when applied on carefully selected patients. In general, the less invasive character makes it favourable over surgical treatment. Device placement was overall successful and is rarely complicated by procedure-related complications. Complete occlusion was not always achieved but gross reduction in shunt fraction led to good results regarding mortality and post-intervention haemodynamic stability, thus proving its efficacy. Following diagnosis, when percutaneous occlusion was applied in the acute phase (<14 days) mortality rates varied strongly from 42 % to 100 %, despite high procedural success (50–86.2 %). Better results were obtained with treatment in the chronic phase (≥14 days), reducing the mortality rates to 20–38.9 %. The most important risk factors concerning mortality were the presence of cardiogenic shock and treatment in the acute phase. Further research with a larger patient populations is needed to assess the true value of percutaneous occlusion in post-AMI VSR.
